# APC ameliorates idiopathic membranous nephropathy by affecting podocyte apoptosis through the ERK1/2/YB-1/PLA2R1 axis

**DOI:** 10.1007/s11010-022-04650-7

**Published:** 2023-01-02

**Authors:** Ben Ke, Wen Shen, Yunfei Liao, Jing Hu, Weiping Tu, Xiangdong Fang

**Affiliations:** 1 Department of Nephrology, The Second Affiliated Hospital of Nanchang University, No. 1, Minde Road, Nanchang, 330006 Jiangxi Province People’s Republic of China; 2 Department of Cardiovascular Medicine, The Second Affiliated Hospital to Nanchang University, Nanchang, 330006 Jiangxi Province People’s Republic of China; 3 Department of Cardiovascular Surgery, The Second Affiliated Hospital to Nanchang University, Nanchang, 330006 Jiangxi Province People’s Republic of China; 4 Department of Anesthesia, The Third Hospital of Nanchang, Nanchang, 330006 Jiangxi Province People’s Republic of China

**Keywords:** APC, ERK1/2, YB-1, PLA2R1, Idiopathic membranous nephropathy

## Abstract

Idiopathic membranous nephropathy (IMN) belongs to an important pathogenic category of adult nephrotic syndrome. PLA2R1 exposure is critical for triggering the pathogenesis of PLA2R1-related IMN. However, the pathogenesis of IMN and the molecular mechanism of treatment remain to be further clarified. The expression changes of activated protein C (APC) and PLA2R1 in IMN patients were quantified by qPCR. A zymosan activated serum (ZAS)-induced IMN podocyte model was established in vitro. Podocyte apoptosis was detected via flow cytometry and caspase‑3 assay. The expression levels of APC, p-ERK1/2, ERK1/2, YB-1 and PLA2R1 were detected by western blotting. The regulation relationship between YB-1 and PLA2R1 was detected by dual fluorescent reporter system. In IMN patients, the expression level of PLA2R1 was increased, whereas the expression level of APC was decreased. When APC was added to podocytes in vitro, the phosphorylation of ERK1/2 was increased, which could promote the translocation of YB-1 to the nucleus that reduces the expression of PLA2R1 at the cellular transcriptional level, thereby inhibiting podocyte apoptosis. Our study is the first to report that APC can improve membranous nephropathy by affecting podocyte apoptosis through the ERK1/2/YB-1/PLA2R1 axis. This study will provide a new targeted therapy for IMN patients with high PLA2R1 expression.

## Introduction

Idiopathic membranous nephropathy (IMN) belongs to an important pathogenic category of adult nephrotic syndrome, caused by autoimmune reactions, accounting for about 25% to 40% of adult nephrotic syndrome [1–3]. The onset of idiopathic membranous nephropathy is slow and the clinical symptoms are different. The main manifestations are: massive proteinuria, hypoalbuminemia, hyperlipidemia, and hyperedema. About 1/3 of the patients can be complicated by deep vein thrombosis, low immune function, susceptible to infection. IMN is caused by podocyte-targeted IgG, of which IgG4 is thought to be the predominant form [3, 4]. Podocyte autoantigens recognized in adult IMN contain the M-type receptor for secreted phospholipase A2 (PLA2R1) [5], thrombospondin type 1 domain-containing 7A (THSD7A) [6], and neuroepidermal growth factor-like 1 protein (NELL1) [7]. In addition, the measurement of anti-PLA2R1 antibody in peripheral blood have been widely used as a biomarker for clinical diagnosis, prediction and treatment guidance [8]. Previous studies have shown that PLA2R1 exposure is essential for touching off the pathogenesis of PLA2R1-related IMN [9]. However, the pathogenesis of IMN and the molecular mechanism of treatment remain to be further clarified.

PLA2R1 is one of the mannose receptor family, which belongs to the type I transmembrane receptor and is localized on podocytes [10]. PLA2R1 is a specific receptor that facilitates the phospholipase A2 (sPLA2) internalization, and multiple research groups have found that the physiological role of PLA2R1 in the cells in which it is expressed is related to sPLA2s [11]. Interestingly, the oxidative extracellular milieu might cause PLA2R1 to establish or retain disulfide bonds, leading to long-term expression of pathogenic epitopes especially bound by antibodies against PLA2R1 circulate in peripheral blood of patients with IMN [9, 12]. Current study has shown that miR-130a-5p inhibits angiotensin II-induced podocyte apoptosis via regulating PLA2R1 [13]. PLA2R1 has been determined as the primary target antigen that triggers the cumulation of circulating autoantibodies in over 75% of IMN patients [5]. Previous findings have shown that reducing activation of PLA2R1 and inhibiting PI3K/AKT/mTOR signaling in PLA2R1-activated podocytes helped prevent podocyte apoptosis [14]. However, whether the podocyte membrane antigen PLA2R1 is regulated by other signaling pathways in the pathogenic autoimmune response is currently unclear.

Protein C is a plasma serine protease zymogen in its active form as activated protein C (APC) with great anticoagulant activity. APC exerts pleiotropic cytoprotective properties, including altered gene expression profiles, anti-inflammatory and anti-apoptotic effects, and stabilization of the endothelial and epithelial barrier [15, 16]. Recent studies have identified anti-inflammatory effects of APC in vascular endothelial cells, and APC is involved in regulatory crosstalk of EPCR, ERK, and NF-κB, thereby disrupting TNF signaling [17]. Further, previous data suggest that APC through PAR1/EPCR signaling maintain YB-1 levels by preventing YB-1 ubiquitination through OTUB1 [18]. Moreover, previous studies have shown that APC can improve diabetic nephropathy [19]. However, the molecular mechanism by which APC regulates YB-1 and whether it can improve IMN symptoms remains unknown.

Y-box-binding protein 1 (YB-1) is one of RNA-binding proteins involved in a variety of cellular processes such as RNA splicing, DNA damage repair and stress responses [20, 21]. Under healthy conditions, YB-1 is mainly found in the cytoplasm and plays a key role in regulating various aspects of RNA biology, but under conditions of genotoxic stress, YB-1 translocates to the nucleus, where activation affects immune response, apoptosis and tumor growth [20]. Previous study has shown that YB-1 can target the MEF2B promoter region to inhibit its expression and improve diabetic cardiomyopathy [18]. The role of YB-1 in calcineurin inhibitor-induced nephropathy was able to increase glomerular fibrosis but reduce interstitial fibrosis in the kidney [22]. Whether YB-1 regulates the expression of PLA2R1 in the ZAS-induced IMN model is still unknown.

Here, we reported the expression level of PLA2R1and APC in IMN patients. In our established ZAS-induced IMN cell model, the PLA2R1 expression was reduced through the ERK1/2/YB-1 pathway. Thereby inhibiting podocyte apoptosis. Therefore, our study is the first to report that APC can ameliorate membranous nephropathy by affecting podocyte apoptosis through the ERK1/2/YB-1/PLA2R1 axis.

## Material and methods

### Clinical specimens

All samples were obtained from patients at the Second Affiliated Hospital of Nanchang University with informed written consent and ethical approval granted by the Ethics Committee of the Second Affiliated Hospital of Nanchang University (Ethics approval number: [2019] No.111). The study was conducted in compliance with the International Ethical Guidelines for Research Involving Human Subjects of the Declaration of Helsinki. Kidney biopsies were performed with an 18-gauge crude needle to identify patients with primary membranous nephropathy by collecting samples from patients with positive anti-PLA2R1 antibodies in their blood. Normal kidney tissue from adult living kidney transplant donors was also collected as a control. Urine was collected from patients and normal people, and urine protein concentration was measured using a non-interfering protein assay kit (Calbiochem), and 24 h urine protein volume was calculated based on urine protein concentration and urine volume.

### Cell culture

The human podocyte cell line was obtained from ATCC. Cells were grown in RPMI-1640 medium including 10% fetal bovine serum (Gibco), penicillin–streptomycin (Gibco), 1 mM l-glutamine and insulin, transferrin and selenium (Invitrogen) in 5% CO_2_ at 37 °C.

### Cell treatment

To establish an in vitro podocyte model of IMN, Normal human serum was treated with Zymosan (Sigma) as previously described [23, 24]. Zymosan- activated human serum (ZAS) was used to assemble C5b-9. Human podocytes were treated for 1, 4, 8, 12, and 24 h in medium containing ZAS at a final concentration of 100 μL/mL, and heat-inactivated human serum (HIS) served as a control. For APC treatment experiments, human APC was purchased from Beijing Aslaier Biotechnology Co., Ltd., and human podocytes were treated with different APC concentrations of 1 nM, 2 nM, 10 nM and 20 nM for 30 min. To inhibit ERK1/2 activity, podocytes were treated with SCH772984 (selleckchem), a specific inhibitor of ERK1/2.

### Nucleus and cytoplasm separation experiment

Podocytes were harvested from cell culture dishes, rinsed in PBS and resuspended in hypotonic lysis buffer (10 mM HEPES pH 7.9; 1.5 mM MgCl_2_; 10 mM KCl; 0.5 mM dithiothreitol (DTT)) containing protease inhibitors. Cells were placed on ice for 30 min and then lysed through a 26-gauge needle. Optical microscopy confirmed that cell lysis reached 80–90% of the cell membrane was disrupted after centrifugation at 1,000 rcf for 5 min at 4 °C. The supernatant was collected and labeled as the cytoplasmic fraction. It was then washed once more in lysis buffer and resuspended in high-salt buffer (20 mM HEPES pH 7.9; 420 mM NaCl; 2 mM EDTA pH 8.0; 1.5 mM MgCl_2_; 25% glycerol; 0.5 mM DTT) containing protease inhibitors. After 30 min on ice, the pellet was centrifuged at 10,000 rcf at 4 °C. This nuclear pellet was again resuspended in high-salt buffer containing 1% Triton X-100 and 0.5% SDS, briefly sonicated, and representative of the nuclear fraction.

### Plasmid construction

The knockdown shRNA of YB-1 was constructed into the pLKO.1 puro vector (addgene, 8453). For plasmids overexpressing YB-1 and PLA2R1, the cDNA sequences of human YB-1 and PLA2R1 were ligated into pCMV-M1 vectors (addgene, 23,007), respectively.

### ELISA assay

The levels of PLA2R1 in the serum of patients with kidney disease were determined by enzyme-linked immunosorbent assay (ELISA) using reagents purchased from Wuhan Fine Biotech (EH2051) according to the experimental instructions. Briefly, patient serum samples were collected and added to a 96-well plate, then 100 μL of biotin antibody was added to each well and incubated for 1 h at 37 °C. Then 90 μL of TMB substrate was added to each well and incubated for 30 min at 37 °C protected from light. Finally, 50 μL of termination solution was added to each well and read immediately at 450 nm for analysis and statistics.

### Immunofluorescence

Podocytes were fixed with 10% neutral buffered formalin, followed by blocking and permeabilization with 5% goat serum and 0.1% Triton X-100, and incubated overnight at 4 °C with antibodies against YB-1. DNA was then stained with 1 μg/mL 4',6-diamidino-2-phenylindole (DAPI). Goat anti-rabbit Alexa 488 (Thermo Fisher Scientific) was used as a secondary antibody. Observations were performed by LSM 810 confocal microscope (Zeiss, Germany).

### Western blot

Podocytes were lysed and homogenized in RIPA buffer for 30 min on ice, then centrifuged at 12,000 rpm for 30 min at 4 °C. The supernatant was aspirated and added to 5 × protein loading buffer. The proteins were separated by SDS-PAGE gel electrophoresis and transferred to on a polyvinylidene fluoride membrane (Millipore). Membranes were blocked with 5% nonfat dry milk in TBST buffer for 1 h at room temperature and then incubated with primary antibody overnight at 4 °C. Blots were incubated with the corresponding primary antibodies. The primary antibodies used are as follows: APC antibody (ab40778, Abcam), PLA2R1 antibody (NBP1-84449, Novus Biologicals), β-Actin antibody (4967, Cell Signaling), p-ERK1/2 antibody (9101, Cell Signaling), ERK1/2 antibody (4695, Cell Signaling), YB-1 antibody (4202, Cell Signaling), Lamin B1 antibody (ab65986, Abcam). The next day was incubated with the corresponding secondary antibody. ECL exposure solution was used to visualize Blots (Thermo Fisher Scientific).

### qPCR

Total RNA from podocytes was extracted with the MiRNeasy Mini Kit (Qiagen), followed by first-strand cDNA synthesis using the High-Capacity RNA-to-cDNA Kit (Applied Biosystems). The ABI QuantStudio 5 (Applied Biosystems) system was used to measure the fluorescence intensity of real-time PCR reactions using Power-SYBR green PCR Master Mix (Applied Biosystems). Relative quantities of genes were computed by the 2^–∆∆CT^ method and normalized to β-actin. The corresponding primers are shown below:

- APC: F: 5’-AAGCATGAAACCGGCTCACAT-3’,
- R: 5’-CATTCGTGTAGTTGAACCCTGA-3’;
- YB-1: F: 5’-GGGGACAAGAAGGTCATCGC-3’,
- R: 5’-CGAAGGTACTTCCTGGGGTTA-3’;
- PLA2R1: F: 5’-GCATACAATCAAAGGGAACACCC-3’,
- R: 5’-TCGTGGCACACCACAGTAAG-3’;
- Β-actin: F: 5’-CTCCATCCTGGCCTCGCTGT-3’,
- R: 5’-GCTGTCACCTTCACCGTTCC-3’.

### Dual-luciferase activity assay

Cells were harvested 48 h after transfection. The experimental steps were performed according to the operating manual of the dual-luciferase reporter assay kit (Promega). The harvested cells were lysed on ice with lysis buffer, and the supernatant was collected by centrifugation at 4 °C for the next step of detection. Luciferase activity was detected using a luminometer (Promega). Renilla and firefly luciferase activities were measured in each sample and the ratio between them was calculated.

### Flow cytometry

The ZAS-induced IMN model and the apoptosis of podocytes under different treatment conditions were detected. First, the podocytes grown in log phase were seeded in 6-well plates at 1 × 10^5^ cells per well, and after culturing for 48 h, the podocytes were collected by trypsinization, washed three times with pre-cooled PBS, and then added with Annexin V-FITC binding solution. Resuspend cells gently, add Annexin V-FITC and propidium iodide staining solution, and mix gently. Finally, cells were incubated at room temperature in the dark for 20–30 min before analysis with a BD FACSLyric™ Clinical flow cytometer.

### TUNEL assay

Cells were seeded onto 6-well plates, separated out, and cytospanned on slides. Cells were then fixed, permeabilized, and labeled according to the protocol of the TUNEL BrightRed Apoptosis Detection Kit (Vazyme). Apoptotic podocytes were determined by terminal deoxynucleotidyl transferase-mediated DUTP nick labeling (TUNEL). Nuclear counterstaining was performed with DAPI (Thermo Fisher Scientific). Images were captured under a fluorescent microscope. The ratio between apoptotic cells and nuclei was used to calculate the percentage of apoptotic cells.

### Caspase‑3 assay

Podocytes were harvested and lysed with cell lysis buffer for 15 min on ice, followed by centrifugation at 20,000×*g* for 15 min at 4 °C. Transfer the supernatant to a pre-chilled centrifuge tube before assaying for caspase-3 activity. AC-DevD-PNA (2 mM; Beyotime) was added to the samples, followed by incubation at 37 °C for 2 h. To obtain the results, the absorbance value of each sample at 405 nm wavelength was measured using an enzyme label (ThermoFisher Scientific).

### Statistical analysis

Each experiment was performed with at least 3 independent replicates and results were presented as mean ± SEM. Statistical analysis of data and presentation of results was performed using GraphPad Prism 9.0. The data between groups were compared by one-way analysis of variance (ANOVA) or a two-tailed unpaired Student's t-test, and the difference was statistically significant at P < 0.05.

## Results

### The expression levels of APC and PLA2R1 in clinical and podocyte models of membranous nephropathy

**Fig. 1:**
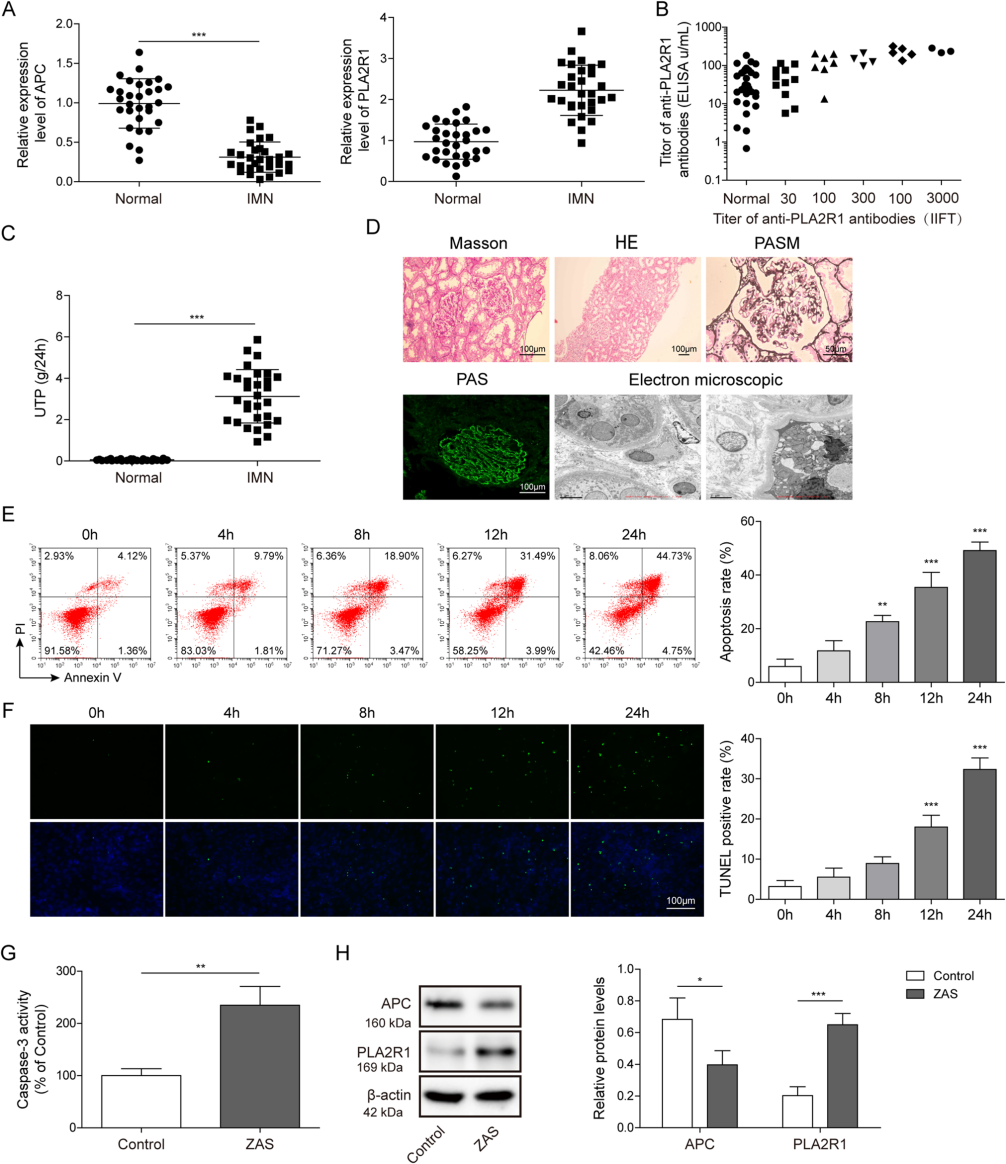
Detection of the expression of APC and PLA2R1 in the clinical level and cell model of IMN. **A** The expression levels of APC and PLA2R1 was detected by qPCR in clinical patients with IMN, *n* = 30 in each group. **B** The protein level of PLA2R1 in serum was detected using ELISA, *n* = 30 in each group. **C** The urine protein levels of IMN patients were tested using a non-interfering protein assay kit, n = 30 in each group. **D** Histomorphological analysis of IMN patients. **E** Cell apoptosis was detected by flow cytometry and the percentage of apoptosis was calculated, *n* = 3 in each group. **F** TUNEL assay for detecting apoptosis in ZAS-induced IMN podocyte model. **G** Caspase-3 activity kit was used to measure Caspase-3 activity in ZAS-induced IMN podocyte model, *n* = 3 in each group. **H** Western blot assay was used to detect the protein expression levels of APC and PLA2R1, *n* = 3 in each group. The measurement data were presented as Mean ± SD was applied to express data. *P < 0.05, **P < 0.01, ***P < 0.001. Scale bars: 100 μm for Masson, HE, PAS staining, and 50 μm for PASM staining. 0.5 μm and 0.2 μm for electron microscope

In order to detect the expression of APC and PLA2R1 in patients with IMN, we randomly selected 30 IMN patients and healthy volunteers. We measured the APC and PLA2R1 expression in the serum of IMN patients by qPCR and found that the expression level of APC decreased while the expression level of PLA2R1 increased (Fig. 1A). The protein level of PLA2R1 in serum was detected using ELISA and the results showed that it was also significantly elevated (Fig. 1B). The urine protein levels of IMN patients were tested to be markedly raised (Fig. 1C). To characterize the pathomorphology of patients with membranous nephropathy, we performed histomorphological observation, Masson staining, HE, PASM, PAS immunofluorescence and electron microscopic analysis of the patients' tissues, respectively. The findings revealed vacuolar degeneration of the renal tubular epithelium and inflammatory cell infiltration in the renal interstitium in patients with IMN (Fig. 1D). APC has a protective effect on cell survival, and PLA2R1 has been identified as the main target antigen in patients with IMN. To further study the interaction between APC and PLA2R1 in IMN, we established an IMN model in vitro by adding 100 μL/mL of enzymatically activated serum (ZAS) to the culture medium of human renal podocytes to treat 0, 4, 8, 12 and 24 h. Flow cytometry was used to detect the apoptosis of cells after ZAS treatment, and the results showed that more than 40% of the cells were apoptotic after 24 h of ZAS treatment, the results indicated that ZAS treatment resulted in apoptosis of human renal podocytes (Fig. 1E). In addition, TUNEL assay also indicated that ZAS treatment led to increased apoptosis of human renal podocytes (Fig. 1F). Consistent with our detection of a significant increase in the level of Caspase-3 in human renal podocytes after ZAS treatment (Fig. 1G). Next, we detected the expression levels of APC and PLA2R1 by Western blotting (WB) in the established IMN podocyte model in vitro, and found that APC protein level decreased and PLA2R1 protein level increased after ZAS treatment (Fig. 1H). This indicates that we have successfully established an in vitro model of IMN.

### APC treatment inhibits podocyte apoptosis in ZAS-induced IMN model through increased ERK1/2 phosphorylation

**Fig. 2:**
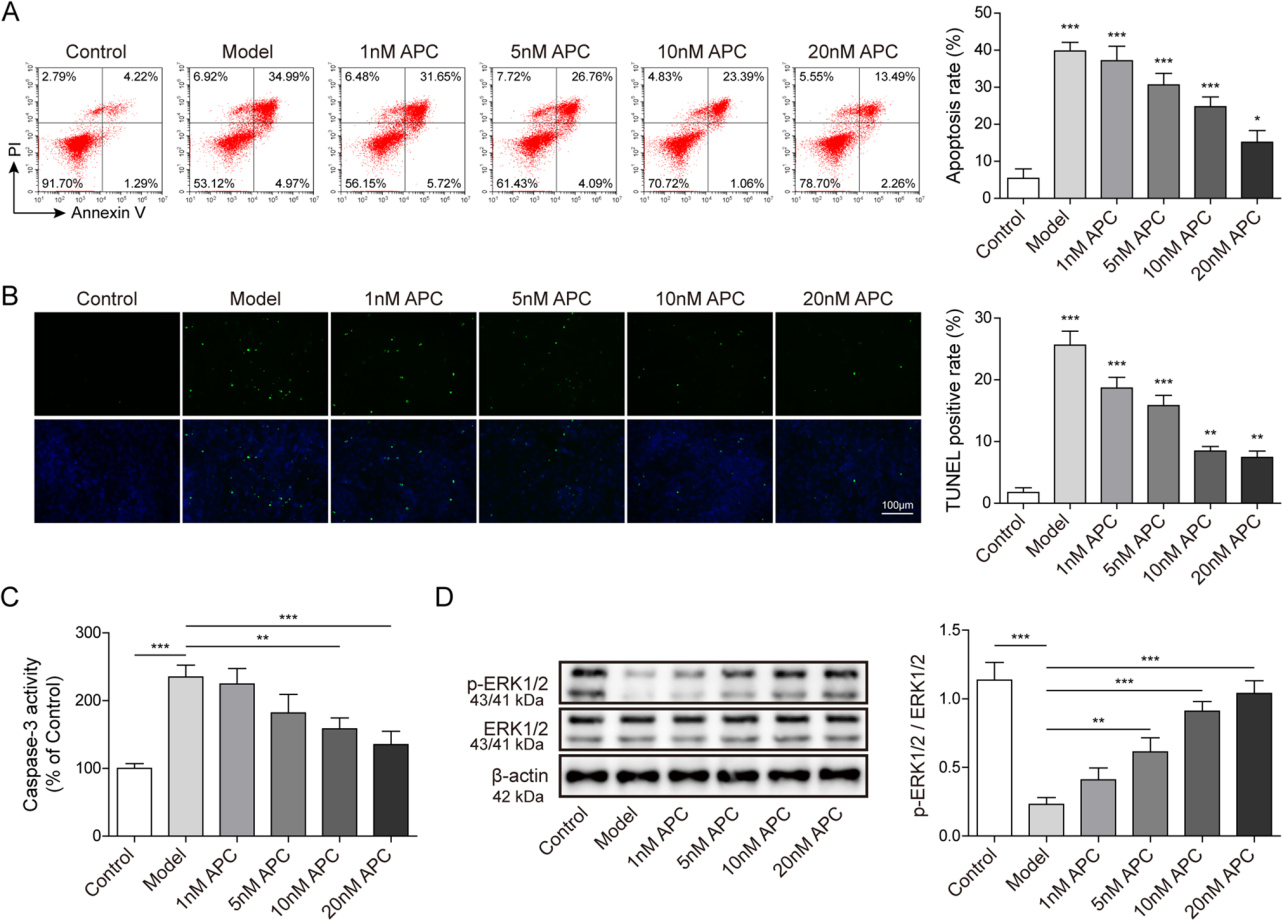
APC inhibits ZAS-induced podocyte apoptosis through ERK1/2 pathway. **A** Cell apoptosis was detected by flow cytometry after different concentrations of APC and the percentage of apoptosis was calculated. **B** TUNEL assay was performed to detect apoptosis in the APC-treated IMN podocyte model. **C** The Caspase-3 activity kit was used to measure the Caspase-3 activity in the IMN podocyte model after different APC treatment concentrations. **D** Western blot assay was used to detect the expression levels of ERK1/2 and p-ERK1/2 in the IMN podocyte model after different APC concentrations. The measurement data were presented as Mean ± SD was applied to express data. *n* = 3, *P < 0.05, **P < 0.01, ***P < 0.001. Scale bars: 100 μm for TUNEL

What is the correlation between the low expression APC and apoptosis in IMN patients. Whether increasing the level of APC will inhibit the apoptosis of podocytes. To answer these questions, we added different concentrations of APC to the in vitro IMN model (100 μL/mL ZAS treated human renal podocytes for 24 h) to observe the effect on podocyte apoptosis. The control group was normal human renal podocytes without ZAS induction. We found that the apoptosis rate of podocytes decreased gradually with the increase of APC concentration (Fig. 2A). Besides, TUNEL assay also indicated that the increase of APC concentration treatment led to decreased apoptosis of IMN model (Fig. 2B). Likewise, the activity level of Caspase-3 was significantly reduced after APC treatment (Fig. 2C). These results suggest that APC can inhibit apoptosis in an in vitro IMN cell model. Previous studies have found that APC can activate the phosphorylation activity of ERK1/2 to regulate a series of biological functions in tumors, acute pancreatitis and vascular endothelial cells [17, 25, 26]. We detected ERK1/2 phosphorylation levels with specific ERK1/2 phosphorylation antibody and found that the phosphorylation levels of ERK1/2 were significantly increased in podocytes with increasing concentrations of APC (Fig. 2D). Thus, we reported that increasing the concentration of APC can activate the activity of ERK1/2 to inhibit ZAS-induced apoptosis of podocytes in vitro.

### ERK1/2 ameliorates IMN by promoting YB-1 entry into the nucleus in a podocyte model

**Fig. 3:**
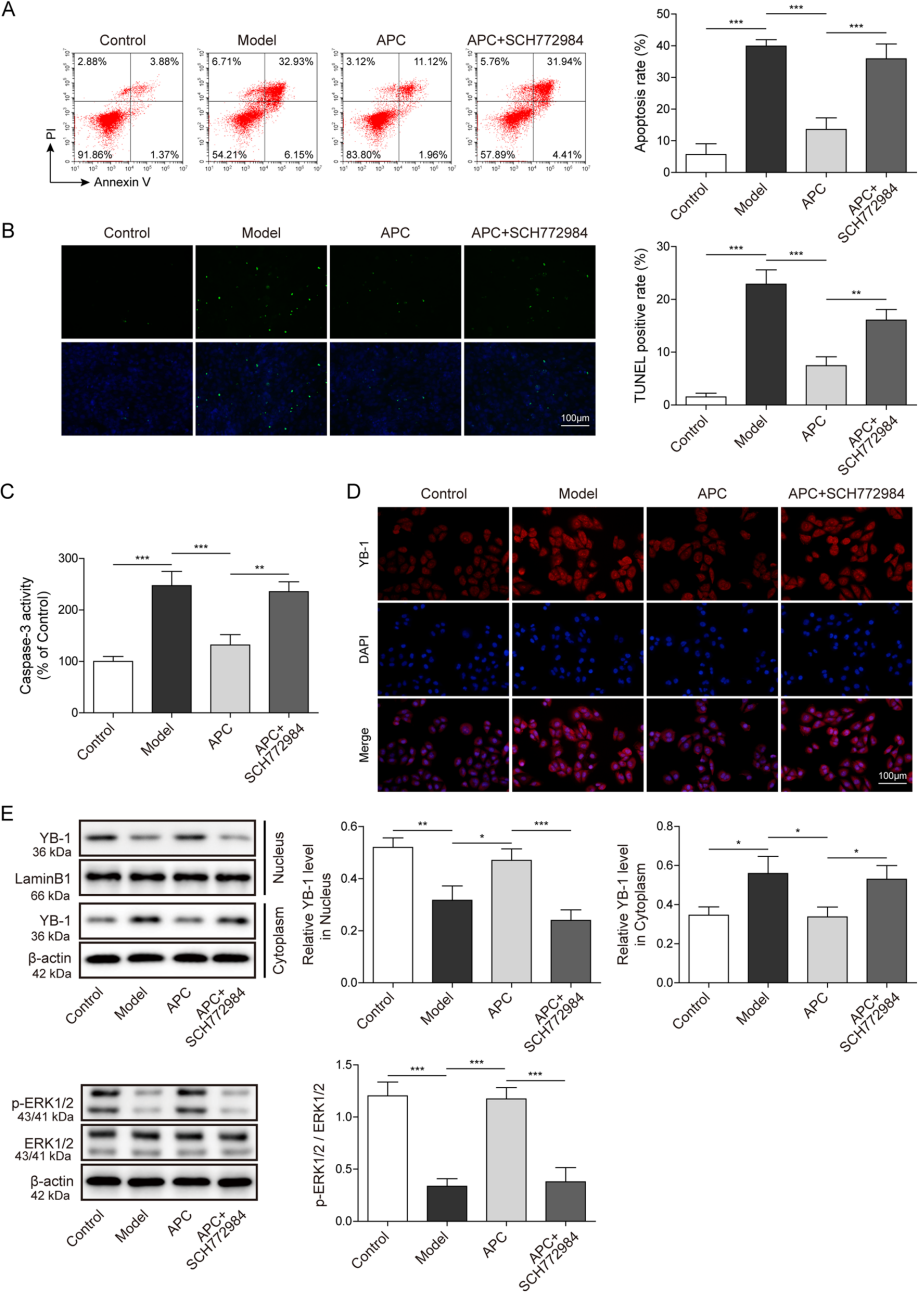
ERK1/2 can promote the translocation of YB-1 to the nucleus and inhibit ZAS-induced podocyte apoptosis. **A** Cell apoptosis was detected after APC and SCH772984 treatment by flow cytometry, and the percentage of apoptosis was calculated. **B** TUNEL assay was performed to detect apoptosis in the IMN podocyte model. **C** Caspase-3 activity kit was used to measure Caspase-3 activity in IMN podocyte model after APC and SCH772984 treatment. **D** Immunofluorescence was used to measure YB-1. **E** The expression levels of ERK1/2, p-ERK1/2 and YB-1 in the nucleus and cytoplasm of the IMN podocyte model after APC and SCH772984 treatment were detected by western blotting. The measurement data were presented as Mean ± SD was applied to express data. *n* = 3, *P < 0.05, **P < 0.01, ***P < 0.001. Scale bars: 100 μm for TUNEL and immunofluorescence

To further study the direct relationship between the activity of ERK1/2 and the apoptosis of IMN in vitro cell model, we treated the cells with ERK1/2 inhibitor SCH772984. We found that the inhibitory effect of APC on apoptosis of podocytes was reversed by SCH772984 after adding APC and SCH772984 at the same time (Fig. 3A). Moreover, TUNEL assay also displayed increased apoptosis in the IMN model after the addition of SCH772984 (Fig. 3B). The activity level of Caspase-3 was significantly increased after the simultaneous treatment of APC and SCH772984 (Fig. 3C). These indicate that APC activates the activity of ERK1/2 to suppress apoptosis in IMN, and the inhibition of ERK1/2 activity has an impact on the blocking effect of APC on apoptosis. Recent study has shown that APC can promote YB-1 translocating into the nucleus through ERK1/2 activation [18, 27]. The results of WB showed that YB-1 had obvious entry into the nucleus in the APC-treated IMN cell model. Add both APC and SCH772984 to the IMN podocyte model significantly reduced ERK1/2 phosphorylation and prevented the entry of YB-1 into the nucleus (Fig. 3D). Furthermore, the nuclear localization of YB-1 was visualized by immunofluorescence, and the signal of YB-1 in the nucleus became clearly stronger after APC treatment, and the nuclear localization signal of YB-1 diminished when it was then treated with SCH772984 (Fig. 3E). Collectively, in the IMN podocyte model, ERK1/2 prevented cell apoptosis by promoting the entry of YB-1 into the nucleus.

### YB-1 suppresses PLA2R1 transcription to impede ZAS-induced apoptosis of podocytes

**Fig. 4:**
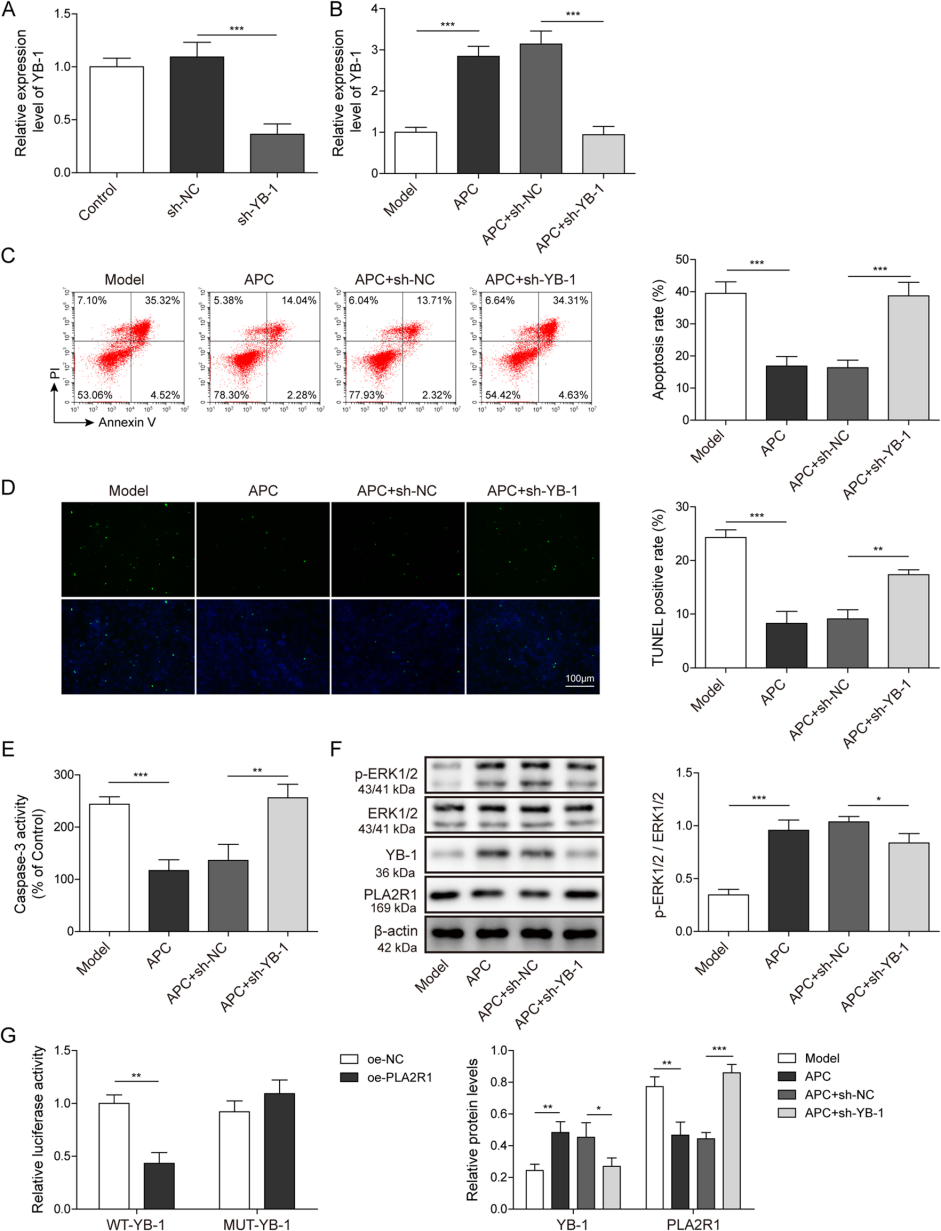
YB-1 can suppress the transcription of PLA2R1 and inhibit ZAS-induced apoptosis of podocytes. **A** The knockdown efficiency of shRNA-YB-1 was detected by qPCR. **B** YB-1 was detected by qPCR after APC treatment. **C** Cell apoptosis was detected after APC treatment and YB-1 knockdown by flow cytometry, and the percentage of apoptosis was calculated. **D** TUNEL assay was performed to detect apoptosis in the IMN podocyte model. **E** The Caspase-3 activity kit was used to measure the Caspase-3 activity in the IMN podocyte model after APC treatment and YB-1 knockdown. **F** The expression levels of YB-1, PLA2R1, ERK1/2 and p-ERK1/2 in the IMN podocyte model after APC treatment and YB-1 knockdown were detected by western blotting. **G** The relationship between YB-1 and PLA2R1 was detected by dual-luciferase reporter system. The measurement data were presented as Mean ± SD was applied to express data. *n* = 3, *P < 0.05, **P < 0.01, ***P < 0.001. Scale bars: 100 μm for TUNEL

To ascertain the effect of YB-1 in IMN, we employed shRNA to knockdown YB-1 in an APC-treated IMN podocyte model. We first examined the knockdown efficiency of YB-1 by qPCR, and the results showed that more than 70% of YB-1 transcription was abolished (Fig. 4A). We tested the apparently high expression of YB-1 in the APC-treated IMN model by qPCR (Fig. 4B). Flow cytometry was used to detect cell apoptosis after YB-1 knockdown in APC-treated IMN podocyte model, and it was found that cell apoptosis was significantly increased (Fig. 4C). In addition, TUNEL testing displayed increased apoptotic signaling from YB-1 knockdown in APC-treated IMN podocyte models (Fig. 4D). Consistently, Caspase-3 activity was also significantly elevated compared to APC-treated and sh-NC controls (Fig. 4E). Therefore, inhibition of YB-1 expression could enhance apoptosis in APC-treated IMN podocyte model. PLA2R1 has been identified as the major target antigen in IMN patients. In this study, we also found the expression level of PLA2R1 was increased in IMN clinical samples (Fig. 1A). The expression of YB-1 is restricted in IMN, whereas the expression of PLA2R1 is up-regulated.

What is the relationship between YB-1 and PLA2R1? To answer this question, we detected the expression levels of the indicated proteins by WB in the APC-treated and YB-1-knockdown IMN podocyte model, and found that the expression of YB-1 was decreased, and the expression of PLA2R1 was significantly increased compared with the APC- and sh-NC-treated cells. In contrast, the phosphorylation levels of ERK1/2 did not change (Fig. 4F). The results of the dual-luciferase reporter system showed that fluorescent expression was downregulated in the absence of the mutated promoter. Therefore YB-1 inhibits the expression of PLA2R1 (Fig. 4G). Taken together, YB-1 inhibits APC-induced apoptosis in podocytes by repressing the transcription of PLA2R1.

### APC ameliorates ZAS-induced apoptosis of podocytes via the ERK1/2/YB-1/PLA2R1 axis

**Fig. 5:**
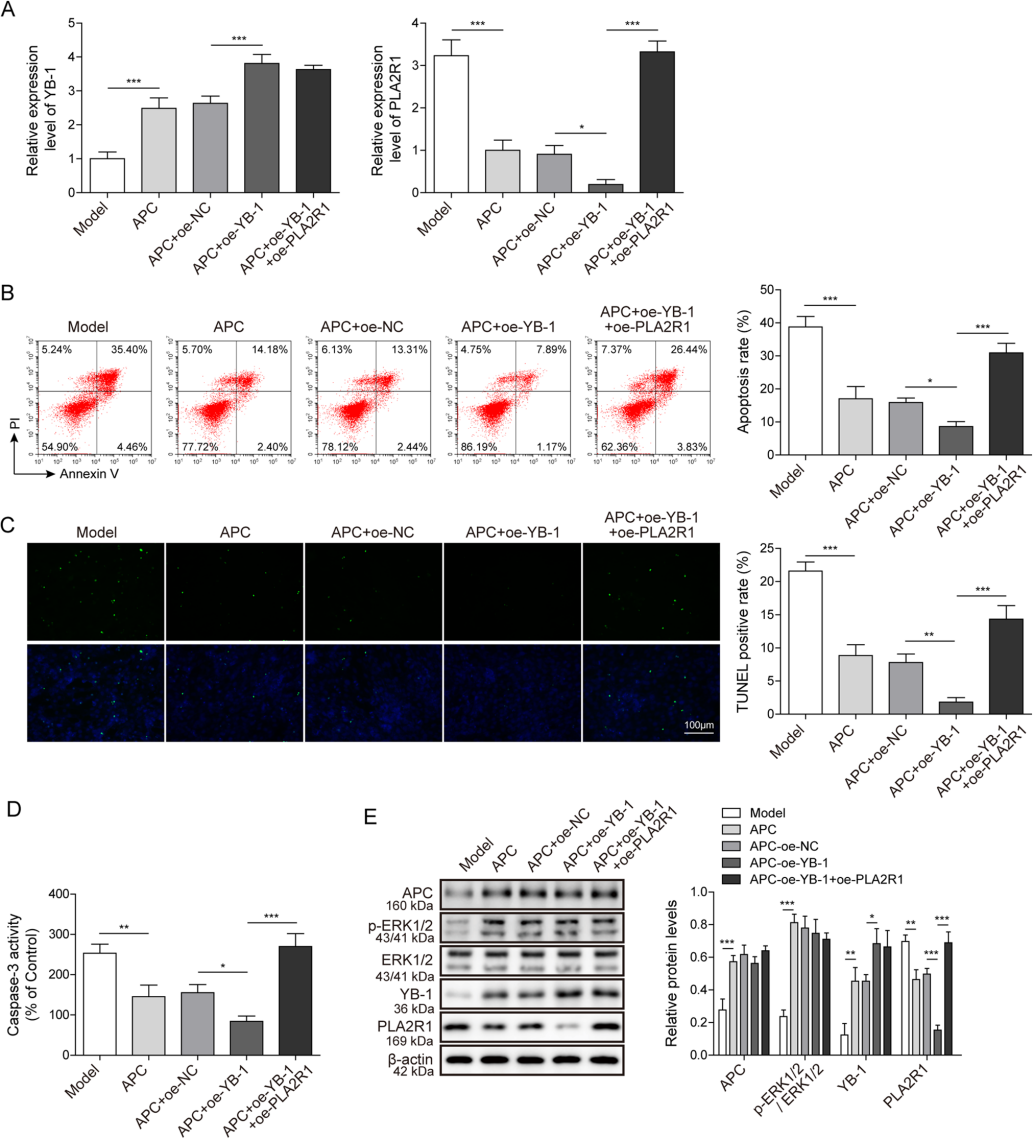
APC improves ZAS-induced podocyte apoptosis through ERK1/2/YB-1/PLA2R1 axis. **A** The expression levels of YB-1 and PLA2R1 were detected by qPCR. **B** Cell apoptosis was detected after overexpression of YB-1 and PLA2R1 by flow cytometry, and the percentage of apoptosis was calculated. **C.** TUNEL assay was performed to detect apoptosis in the IMN podocyte model. **D** Caspase-3 activity kit was used to measure Caspase-3 activity in IMN podocyte model after overexpression of YB-1 and PLA2R1. **E** The expression levels of APC, p-ERK1/2, ERK1/2, YB-1 and PLA2R1 were detected by western blotting in the IMN podocyte model after overexpression of YB-1 and PLA2R1. The measurement data were presented as Mean ± SD was applied to express data. *n* = 3, *P < 0.05, **P < 0.01, ***P < 0.001. Scale bars: 100 μm for TUNEL

To figure out the regulatory network between APC and PLA2R1 in the ZAS-induced podocyte IMN model, we simultaneously treated with APC and overexpressed YB-1 (oe-YB-1) and PLA2R1 (oe-PLA2R1) in this model. The expression levels of YB-1 and PLA2R1 were detected by qPCR, and it was found that in APC-treated and overexpressed YB-1, the YB-1 expression level was increased, and the PLA2R1 expression level was decreased. In addition, overexpression of PLA2R1 can reverse the expression level of PLA2R1 without changing the expression of YB-1 (Fig. 5A). Flow cytometry was used to detect the apoptosis of different treatment groups. The results showed that the apoptosis was significantly reduced in the APC + oe-YB-1 experimental group, while that in the APC + oe-YB-1 + oe-PLA2R1 experimental group was markedly increased (Fig. 5B). These findings were also consistent with the TUNEL testing results (Fig. 5C). Similarly, the activity of Caspase-3 showed the same trend as apoptosis (Fig. 5D). The previous experimental results showed that the protein levels of p-ERK1/2 and YB-1 were both up-regulated in the APC-treated IMN podocyte model. Next, we used WB to detect the protein levels in the cells of the APC + oe-YB-1 and APC + oe-NC experimental groups and found that the protein level of APC and p-ERK1/2 remained unchanged, but the protein expression levels of PLA2R1 was decreased. Compared with the APC + oe-YB-1 group, the APC + of-YB-1 + oe-PLA2R1 treatment group had no change in the intracellular protein expression levels of APC, YB-1 and p-ERK1/2, only the PLA2R1 protein expression level increased (Fig. 5E). The results illustrate that APC ameliorated ZAS-induced apoptosis of podocytes through the ERK1/2/YB-1/PLA2R1 axis.

## Discussion

Over the past decade, the understanding of IMN pathogenesis has greatly improved. The discovery of PLA2R1 in 2009 and THSD7A in 2014 as two major autoantigens in IMN has greatly advanced the understanding of molecular pathogenesis [5, 28]. These significant findings have been rapidly applied to clinical diagnosis and treatment monitoring, and anti-PLA2R1 antibodies can be detected in 70%–80% of IMN patients. Anti-PLA2R1 antibody titers are key biomarkers reflecting disease status, prognostic effects, and treatment effects [29, 30]. PLA2R1 is highly expressed in the kidney, which is also able to be released into the systemic circulation to trigger an autoimmune response [31]. In this study, we found that the expression level of PLA2R1 was increased, while the expression level of APC was decreased in IMN patients. In our established ZAS-induced IMN cell model, the addition of APC treatment can increase the phosphorylation of ERK1/2, promote the translocation of YB-1 to the nucleus, and reduce the expression of PLA2R1, thereby inhibiting cell apoptosis. Thus, our study is the first to report that APC can improve membranous nephropathy by affecting podocyte apoptosis through the ERK1/2/YB-1/PLA2R1 axis.

We also found that APC was at a remarkably low expression level in IMN patients, and the expression of APC was also repressed and cell apoptosis was increased in a pathogenic model of ZAS-induced IMN. Previous reports have shown that APC exerts pleiotropic cytoprotective properties, including altered gene expression profiles, anti-apoptotic effects and anti-inflammatory [15, 16]. We hypothesize that the low expression level of APC in IMN did not have sufficient protective effect on podocytes and contributed to the increase of podocyte apoptosis. Therefore, we added APC to treat IMN model cells and detected that the apoptosis of podocytes was hindered. This is consistent with previous studies that APC can improve diabetic nephropathy [19]. Recent studies have identified the anti-inflammatory effects of APC in vascular endothelial cells, and APC is involved in regulatory crosstalk of EPCR, ERK, and NF-κB, thereby disrupting TNF signaling [17]. Further, previous data suggest that APC through PAR1/EPCR signaling maintain YB-1 levels by preventing YB-1 ubiquitination through OTUB1 [18, 27]. Here, adding APC to IMN cells can increase the phosphorylation of ERK1/2 and promote the translocation of YB-1 to the nucleus. It was also found that the expression level of YB-1 in nucleus was seriously reduced using SCH772984 to block the activity of ERK1/2. Taken together, APC can promote YB-1 into the nucleus through phosphorylation of ERK1/2 in IMN.

Interestingly, the translocation of YB-1 to the nucleus was significantly increased in the APC-treated IMN model. As we well-known, under conditions of genotoxic stress, YB-1 translocates to the nucleus where activation affects immune response and apoptosis [32]. Previous study has shown that YB-1 can target the MEF2B promoter region to inhibit its expression and improve diabetic cardiomyopathy [18]. The role of YB-1 in calcineurin inhibitor-induced nephropathy was able to increase glomerular but decrease interstitial fibrosis [22]. According to previous reports, PLA2R1, a constitutively transmembrane expressed receptor in podocytes, has been identified as an autoimmune target of IMN [5, 28]. Current study has shown that MiR-130a-5p inhibits angiotensin II-induced podocyte apoptosis by regulating PLA2R1 [13]. Our results show that YB-1 translocates into nucleus represses PLA2R1 in a ZAS-induced IMN model.

In summary, we successfully established an in vitro ZAS-induced IMN model, in which the expression level of PLA2R1 was up-regulated, while the expression level of APC was down-regulated. When cells were treated with APC, the expression of PLA2R1 was reduced, and the signal transduction mechanism was dependent on phosphorylated ERK1/2 to regulate the entry of YB-1 into the nucleus. This study will provide a new targeted therapy for IMN patients with high PLA2R1 expression, which can block the apoptosis of podocytes by reducing the level of PLA2R1 through APC. The current study has only been validated in an in vitro cell model of IMN, and in the future we will conduct further experiments in an in vivo model of IMN, such as a mouse model, hoping to obtain consistent results.

## Data Availability

Thus, our study is the first to report that APC can ameliorate membranous nephropathy by affecting podocyte apoptosis through the ERK1/2/YB-1/PLA2R1 axis.

## Abbreviations

IMN: Idiopathic membranous nephropathy
APC: Activated protein C
ZAS: Zymosan activated serum
ERK1/2: Extracellular signal-regulated kinases 1/2
YB-1: Y-box-binding protein 1
PLA2R1: M-type receptor for secreted phospholipase A2
NELL1: Neuroepidermal growth factor-like 1 protein
THSD7A: Thrombospondin type 1 domain-containing 7A
PI3K: Phosphoinositide 3-Kinase
AKT: RAC (Rho family)-alpha serine/threonine-protein kinase
mTOR: Mammalian target of rapamycin
EPCR: Endothelial protein C rece
PAR1: Proteinase-activated receptor 1
MEF2B: Myocyte enhancer-binding factor 2B
OTUB1: OTU deubiquitinase, ubiquitin aldehyde binding 1
